# Multi-parameter systematic strategy opinion that predicts, prevents, and personalized treats a cancer

**DOI:** 10.1186/1878-5085-5-S1-A25

**Published:** 2014-02-11

**Authors:** Xianquan Zhan, Rong Hu, Xiaowei Wang

**Affiliations:** 1 Key Laboratory of Cancer Proteomics of Chinese Ministry of Health, Xiangya Hospital, Central South University, 87 Xiangya Road, Changsha, Hunan 410008 P. R. China; 2 Hunan Engineering Laboratory for Structural Biology and Drug Design, Xiangya Hospital, Central South University, 87 Xiangya Road, Changsha, Hunan 410008 P. R. China; 3 State Local Joint Engineering Laboratory for Anticancer Drugs, Xiangya Hospital, Central South University, 87 Xiangya Road, Changsha, Hunan 410008 P. R. China; 4 The State Key Laboratory of Medical Genetics, Central South University, 88 Xiangya Road, Changsha, Hunan 410008 P. R. China

## 

Cancer is a complex whole-body disease that alters in the levels of gene, protein, and metabolite, and that involves multi-factors, multi-processes and multi-consequences. Individual variation is involved in each stage of prediction/prevention, early-stage diagnosis/therapy, and late-stage diagnosis/therapy. The development of omics and systems biology has promoted one to gradually change paradigms in oncology from traditional single factor strategy to multi-parameter systematic strategy. The therapeutic model of cancer has changed from the general radiotherapy and chemotherapy to personalized strategy. The development of PPPM will substantially change the understanding, prediction, prevention, and therapeutic model of cancer from a systematical and comprehensive point of view in the future.
